# Diversity of Mycogenic Oxide and Chalcogenide Nanoparticles: A Review

**DOI:** 10.3390/biomimetics8020224

**Published:** 2023-05-26

**Authors:** Ekaterina A. Loshchinina, Elena P. Vetchinkina, Maria A. Kupryashina

**Affiliations:** Laboratory of Microbiology, Institute of Biochemistry and Physiology of Plants and Microorganisms, Saratov Scientific Centre of the Russian Academy of Sciences (IBPPM RAS), 410049 Saratov, Russia; loshchinina@yandex.ru (E.A.L.); kupryashina_m@mail.ru (M.A.K.)

**Keywords:** biogenic nanoparticles, green synthesis, oxides, chalcogenides, nanoparticle characteristics

## Abstract

Oxide and chalcogenide nanoparticles have great potential for use in biomedicine, engineering, agriculture, environmental protection, and other research fields. The myco-synthesis of nanoparticles with fungal cultures, their metabolites, culture liquids, and mycelial and fruit body extracts is simple, cheap and environmentally friendly. The characteristics of nanoparticles, including their size, shape, homogeneity, stability, physical properties and biological activity, can be tuned by changing the myco-synthesis conditions. This review summarizes the data on the diversity of oxide and chalcogenide nanoparticles produced by various fungal species under different experimental conditions.

## 1. Introduction

Nanotechnology and nanomaterials science are rapidly developing fields, which contribute greatly to the development of modern technology and biomedicine. An important challenge is the development of simple, effective, and cheap methods of producing highly monodispersed, stable, and biocompatible nanoparticles (NPs) with the required chemical composition, shape, size, biological activity, and other properties. The recent increase in attention to environmental safety, natural resource exhaustibility, and human health safety has led to the increasing development of green NP-producing technologies by biosynthesis methods [1,2,3,4]. Owing to its being environmentally benign and less resource-intensive than other methods, the synthesis of nontoxic and biocompatible NPs by using living organisms and a variety of biological materials derived from them is a promising alternative to physical and chemical fabrication methods.

The ability to biosynthesize NPs has been found in many organisms, including animals, plants, bacteria, fungi, actinomycetes, algae, lichens, and viruses [5]. Among this diversity of biological objects used for green NP synthesis, a special place is occupied by fungi [6,7,8]. Fungal cultures produce a wide range of proteins with high enzymatic activity, and due to that they can convert metals and other elements into less toxic forms. This includes the formation of NPs, which then accumulate in large quantities within the mycelium and/or extracellularly. As a result, micro- and macro-mycetes from different taxonomic groups can be successfully used to produce NPs and nanomaterials on an industrial scale. The applications of fungi in myco-nano-synthesis are also highly versatile. NPs with different characteristics can be obtained either by growing fungal cultures on media with precursors or by incubating these precursors with mycelial bio-mass, culture liquid filtrates, extracts from vegetative mycelium, fruiting bodies and other morpho-structures, and purified proteins and other metabolites isolated from fungi [5]. In addition, the properties of biogenic NPs depend on medium composition, chemical composition and concentration of the precursor, incubation time, stirring rate, temperature, pH and other conditions. By varying these, the chemical composition, shape, size, homogeneity, stability, and biological activity of formed particles can be controlled [9,10,11,12].

Fungal cultures can form NPs of various chemical compositions. The myco-synthesis of elementary gold and silver NPs is the most commonly studied so far, whereas a number of other metals and metalloids have been studied to a lesser extent. In addition, fungi can form NPs of a more complex chemical composition, such as oxides and salts. Among the inorganic NPs, metal oxide and chalcogenide (sulfide, selenide, and telluride) NPs are of great interest for multidisciplinary nanotechnology. They can find a wide range of applications owing to their special physical, chemical, and biological properties, which distinguish them from bulk materials of the same chemical composition [13,14].

Oxide NPs are promising for use in nano-catalysis, biosensing, biomedicine, wastewater purification, and removal of heavy metals, dyes, and microbial contaminants [14,15,16,17,18,19]. Applications for chalcogenide NPs include cell imaging, cancer research and therapy, antimicrobials, and energy and optoelectronics applications [13,20,21,22]. An important group of NPs are quantum dots (QDs), a type of small (less than 10 nm) colloidal fluorescent semiconducting nanocrystalline particles composed of group II–VI, III–V, or IV elements [13,23,24]. Owing to their unique structural, optical, electrochemical, and photochemical properties, QDs can be used in numerous technological applications, such as biosensing, bioimaging, photovoltaics, nanomedicine, and drug delivery.

Because of this widespread demand for nanoparticles and nanomaterials of oxides and chalcogenides, the study of their synthesis by fungi has started to develop rapidly in recent years. However, until now, little attention has been paid to the comparison of the properties of nanoparticles of the same compound obtained with different species of fungi and under different conditions, and, consequently, to the optimization of the process to obtain nanoparticles with the desired properties. This review summarizes the current information on the myco-synthesis of nanoparticles of oxides, sulfides, selenides, and tellurides by fungi belonging to different taxonomic groups, the variety of their characteristics under different synthetic conditions, and the potential for their further application.

## 2. Myco-Synthesis of Oxide Nanoparticles

To date, the ability to form elementary metal and metalloid NPs has been found in many fungal species. However, the number of elements that can be sources of mycogenic NPs is rather small and includes gold, silver, platinum, palladium, iron, copper, selenium, and tellurium [5]. For oxides and salts, the range of chemical composition for mycogenic NPs is much wider, yet most of them remain very poorly studied. Among fungi-mediated oxide NPs, titanium, zinc, iron, and copper oxides have been studied to the greatest extent.

### 2.1. Myco-Synthesis of Copper Oxide Nanoparticles

Copper oxide NPs have attracted high attention because copper is one of the most important elements in modern technologies and is readily accessible [25]. Copper oxide is widely used in catalysis, superconductors, thermoelectric and sensing materials, ceramics, gas sensors, and many other fields. Biomedical applications of these NPs include biosensors, cancer medicine, and antimicrobials [26]. In recent years, the fungi-mediated synthesis of copper oxide NPs has become of interest (Table 1).

CuO nanospheres (average size 32.4 nm) with high antimicrobial and antitumor activities were obtained with *Aspergillus flavus* culture liquid [27]. CuO NPs synthesized with *Aspergillus terreus* culture liquid showed anticancer activity in a concentration-dependent manner [30]. Other works showed that CuO nanospheres synthesized with *A. terreus* culture liquid had potent antioxidant and antimicrobial activities [28,29].

Honary et al. studied copper oxide NP synthesis by three *Penicillium* species [35]. *Penicillium aurantiogriseum*, *Penicillium citrinum*, and *Penicillium waksmanii* culture liquids mediated the fabrication of copper oxide nanospheres of various diameters. The effect of several parameters on the particle size and the polydispersity index for the synthesis of NPs under ambient conditions was also investigated. It was concluded that there is a direct correlation among pH value, precursor concentration, polydispersity index, and particle size. Spherical CuO NPs were also obtained with a *Penicillium chrysogenum* culture liquid filtrate with the aid of gamma rays at various doses [31]. The NPs were an active antibacterial agent against fungal and bacterial crop pathogens.

Copper oxide NPs of various sizes and shapes were synthesized with *Pleurotus florida* bio-mass and two different precursor salts [36]. Spherical, partially spherical, and oval particles (22.55–60.09 nm) were formed after the incubation with CuCl_2_, whereas the use of CuSO_4_ resulted in hexagonal and partially spherical NPs (12.82–48.86 nm).

Spherical copper oxide NPs with a size of 10 to 190 nm and an average diameter of 110 nm were synthesized with a cell-free extract of *Trichoderma asperellum* [32]. These NPs induced photo-thermolysis of human lung cancer cells. CuO NPs with a rare morphology were obtained by Consolo and colleagues with a *Trichoderma harzianum* extract [33]. These particles were in the shape of elongated fibers 38–77 nm in width and 135–320 nm in length, and were inhibitory to fungal phytopathogens.

A *Stereum hirsutum* mycelial extract was found to be effective at synthesizing copper NPs [34]. Copper/copper oxide NP fabrication was studied under different pH conditions and in the presence of three copper salts (CuCl_2_, CuSO_4_, and Cu(NO_3_)_2_). Greater NP formation was shown with 5 mM CuCl_2_ under alkaline conditions. The resulting NPs were mainly spherical and had sizes of 5 to 20 nm.

### 2.2. Myco-Synthesis of Iron Oxide Nanoparticles

Iron oxide is a mineral compound that exists in various polymorphic forms, the main of which are hematite (α-Fe_2_O_3_), maghemite (γ-Fe_2_O_3_), and magnetite (Fe_3_O_4_) [15]. Mycogenic NPs of iron oxides (III) and (IV) obtained by different researchers with different biological objects and precursors differ greatly in both size and shape (Table 2). Spherical, cubic, irregular, octahedral, and flakelike iron oxide nanoparticles were obtained with mushroom cultures.

Maghemite (γ-Fe_2_O_3_) nanospheres (15–66 nm) were synthesized with a *Penicillium expansum* mycelial extract filtrate [38]. These NPs were effective biocatalysts for the decolorization and degradation of textile and tanning wastewater effluents in a dose- and time-dependent manner. Spherical hematite (α-Fe_2_O_3_) NPs with a mean size of 207 nm, fabricated with a *T. harzianum* extract, enhanced *T. harzianum* biocontrol activity against the phytopathogen *Sclerotinia sclerotiorum* [37]. Small cubic Fe_2_O_3_ NPs with an average size of 9 nm, active against Gram-positive and Gram-negative bacteria, were obtained with an *Alternaria alternata* mycelial extract [39]. Synthesis of Fe_2_O_3_ nanospheres was also achieved with culture liquids of the manglicolous fungi *T. asperellum*, *Phialemoniopsis ocularis*, *Fusarium incarnatum*, and *Penicillium pimiteouiense* [41,42]. Their size varied depending on the species of fungus used for myco-synthesis. Manglicolous fungi–derived Fe_2_O_3_ NPs were effective in removing Cr (VI) from synthetic wastewater [42].

The fungi *Fusarium oxysporum* and *Verticillium* sp. formed iron oxide NPs predominantly in the magnetite (Fe_3_O_4_) phase [45]. Incubation of *F. oxysporum* bio-mass with precursors yielded quasi-spherical NPs, whereas *Verticillium* sp. produced cubo-octahedral particles. Superparamagnetic Fe_3_O_4_ nanoflakes 20–40 nm in width were fabricated with an *Aspergillus niger* mycelial extract [43]. The NPs were successfully used for Cr (VI) removal from an aqueous solution, which provides an eco-friendly, sustainable, and effective route to heavy metal remediation and wastewater treatment. Fe_3_O_4_ NPs obtained with lyophilized bio-mass of *Fusarium solani* were mostly cubic and spherical, with a mean size of 55.3–84.2 nm [46]. They had bactericidal activity against various bacteria, although it was weaker in comparison with that of silver NPs synthesized by *F. solani* in the same study. Mousa et al. fabricated Fe_3_O_4_ nanospheres with *A. terreus* culture liquid and determined optimal conditions for the maximal NP yield [28,29]. These NPs showed promising antioxidant activity and a potent effect against several plant and human pathogens.

Cubic iron oxide NPs (60–70 nm) were obtained with *Aspergillus japonicus* bio-mass, with K_3_[Fe(CN)_6_] and K_4_[Fe(CN)_6_] as precursors [48]. Incubation of *P. florida* mycelium with FeCl_2_ yielded cubic NPs with an average size of 11.90 to 167.63 nm, whereas the FeSO_4_ precursor produced highly agglomerated nanospheres with an average size of 11.16 to 98.81 nm [36].

Abdeen et al. used a combined method to produce magnetic Fe_3_O_4_ NPs [40]. *A. niger* bio-mass was used for the decomposition of FeSO_4_ and FeCl_3_ to FeS and Fe_2_O_3_, respectively. Then, the FeS and Fe_2_O_3_ NPs were used to produce pure iron and Fe_3_O_4_ nanospheres by a physical method.

Several researchers have used fungal cultures to obtain iron oxide nanocomposite materials with enhanced properties. Fe_3_O_4_ NPs with spherical morphology and size varying from 5 to 22 nm were myco-synthesized with an *Aspergillus tamarii* culture filtrate [44]. The conditions for NP production were optimized, including incubation period, stirring speed and time, temperature, and pH. The resulting Fe_3_O_4_ NPs were impregnated into chitosan beads to produce magnetic nanocomposite for textile wastewater treatment. The modified beads showed a good removal capability with improved stability and regeneration, as compared with the control chitosan beads. Another iron oxide NP–based biomaterial was obtained with a spent substrate for *Agrocybe cylindracea* [49]. The Fe_3_O_4_ NP–coated biomaterial described in that study was effective at Cr (VI) removal from wastewater. Ince et al. developed fungal bio-nanocomposite Fe_3_O_4_ materials effective at metal removal from drinks and wastewater. They synthesized chitosan-coated magnetic Fe_3_O_4_ NPs with an *Agaricus campestris* fruit body extract [50] and alginate-coated superparamagnetic Fe_3_O_4_ NPs with an *Amanita vaginata* fruit body extract [51]. Ultrasmall iron oxide NPs were fabricated with an *Amanita muscaria* fruit body extract [47]. Using these NPs, Ivashchenko and colleagues developed a gel formulation for local anticancer therapy, containing *A. muscaria*–derived iron oxide and silver NPs.

### 2.3. Titanium Oxide NPs

Titanium oxide (TiO_2_) NPs are yet another important NPs with outstanding properties. Three mineral forms of TiO_2_ that are known to occur naturally are anatase, rutile, and brookite. TiO_2_ NPs have a wide range of applications owing to their chemical stability, low toxicity, biocompatibility, and good corrosion resistance [52]. Thanks to their high antibacterial, antifungal, antiviral, and anticancer activity, they are important tools in diagnostics, therapeutics, and drug delivery [53]. Various micro- and macro-mycetes can produce TiO_2_ NPs with their living cultures, extracts, bio-mass, and even spores (Table 3).

The ability to synthesize TiO_2_ NPs was found in various *Aspergillus* species. Spherical and oval 62–74-nm TiO_2_ NPs with antibacterial properties were fabricated with *A. flavus* bio-mass [55]. TiO_2_ NPs of 12–15 nm synthesized with an *A. flavus* mycelial extract demonstrated a strong growth-enhancing effect on mung beans and thus can be used as a plant nutrient fertilizer [56]. TiO_2_ nanospheres (73.58–106.9 nm) generated with an *A. niger* extract were found a potent mosquito larvicidal agent [57]. TiO_2_ NPs produced with an *Aspergillus tubingensis* mycelial extract were cubic and pentameric, with a size of 1.5–30 nm [58].

Bansal et al. showed that spherical titania particles (6–13 nm) can be produced with *F. oxysporum* bio-mass and K_2_TiF_6_ as a precursor [61]. Al-Timini and Sermon showed that NPs can be synthesized on the surfaces of Portobello mushroom spores [54]. Using this approach, they prepared TiO_x_, Ag–TiO_x_, and Au–TiO_x_ NPs. Nanohybrids of these NPs with mushroom spores showed antibacterial and/or antifungal activity.

Rehman et al. fabricated irregularly shaped TiO_2_ NPs (80–120 nm) with *Fomes fomentarius* [59] and *Fomitopsis pinicola* [60] fruit body extracts. Both *F. pinicola* and *F. fomentarius*–mediated NPs were active against Gram-positive and Gram-negative bacteria and had an anticancer effect. Protein-capped NPs of TiO_2_ (5–28 nm) were formed directly from micron-sized TiO_2_ powder (150–250 nm) by incubation with *Humicola* sp. mycelium at 50°C [62]. Fungal processing of these large-sized particles bioleached them and transformed them into NPs of a different phase and shape (brookite (circular-shaped)), as compared with the parent material (anatase (disc-shaped powder)).

Manimaran et al. synthesized TiO_2_ NPs with *Hypsizygus ulmarius*, *Pleurotus djamor*, and *Pleurotus sajor caju* fruit body extracts [63,64,65]. The NPs fabricated with *H. ulmarius* expressed a broad-spectrum antimicrobial effect against pathogenic bacteria and anticancer activity [63]. *Pleurotus*-derived TiO_2_ NPs possessed mosquito larvicidal, antibacterial, and anticancer activities [64,65].

Small mesoporous TiO_2_ NPs (10–12 nm) with outstanding photocatalytic performance were formed when *Sachharomyces cerevisae* yeast cells were cultivated with TiCl_4_ [67]. By incubating an *S. cerevisae* culture with TiO(OH)_2_, nanospheres with an average size of 12.57 nm were fabricated [66]. In another study, baker’s yeast was incubated with TiCl_3_ to produce anatase TiO_2_ nanospheres (average size 6.7 nm) [68]. These mycogenic NPs had prominent antibacterial and antifungal properties and were highly photocatalytic in comparison to commercially available 21-nm TiO_2_ NPs.

TiO_2_ NPs obtained with *Trichoderma viride* culture liquid were spherical, with a size of 60–86.67 nm [70]. Their larvicidal, antifeedant and pupicidal activities against the cultivated crop pest *Helicoverpa armigera* can help in eco-friendly pest management. With a *Tricoderma citrinoviride* mycelial extract, irregular, triangular, pentagonal, spherical, and rod-shaped TiO_2_ NPs were synthesized, whose sizes ranged between 10 and 400 nm [69]. They showed excellent antioxidant potential and antibacterial activity against extremely drug-resistant *Pseudomonas aeruginosa* clinical isolates.

### 2.4. Myco-Synthesis of Zinc Oxide Nanoparticles

Zinc oxide (ZnO) NPs have caught the attention of researchers owing to their extensive biological properties, including antibacterial, antifungal, anticancer, anti-inflammatory, antidiabetic, antioxidant, antiviral, wound healing, and cardioprotective activity [71,72]. Their exceptional optical, electrical, and physiochemical properties make ZnO NPs an excellent option for electronics, optoelectronics, bioimaging, biosensors, drug and gene delivery [72,73]. To date, these NPs have been the most commonly studied of all mycogenic oxide NPs (Table 4). Mycogenic ZnO NPs are mostly spherical or hexagonal.

The formation of ZnO nanospheres with sizes ranging between 13 and 15 nm was studied by using an *Acremonium potronii* mycelial extract [74]. The obtained ZnO NPs showed high photocatalytic activity for the degradation of methylene blue dye. ZnO nanospheres with an average size of 14.48 nm were fabricated with an aqueous extract of *Agarius bisporus* as a reducing agent [75]. These NPs were effective inhibitors of microbially influenced corrosion.

The correlation between the zinc tolerance of soil fungi and their potential for the synthesis of ZnO NPs was examined with 19 fungal isolates from the rhizo-spheric soil of plants naturally growing at a zinc mine area [76]. The *Aspergillus aeneus* isolate had a high zinc tolerance and potential for the extracellular synthesis of ZnO NPs. The result was the synthesis of spherical NPs (100–140 nm) coated with proteins, which served as stabilizers.

Spherical 60–80-nm ZnO NPs synthesized with *Aspergillus fumigatus* culture liquid were an effective antimicrobial agent against Gram-negative and Gram-positive bacteria [77]. An *A. fumigatus* mycelial extract induced the formation of oblate spherical and hexagonal ZnO NPs (1.2–6.8 nm) with plant-growth-promoting properties [78]. Hexagonal ZnO NPs with an average size of 66 nm were synthesized by using crushed powder of *A. niger* [80]. Protein-capped ZnO nanospheres with a particle size of about 10–45 nm, possessing antibacterial activity, were obtained with an *A. terreus* mycelial extract [82]. Incubation of *A. terreus* culture liquid with ZnSO_4_ yielded spherical ZnO NPs (28–63 nm) [81]. ZnO NPs with antioxidant and antimicrobial activities were also synthesized with the culture liquid of *A. terreus* and with ZnC_4_H_6_O_4_ as a precursor [28,29].

Aqueous mycelial extracts of two fungi, *A. niger* and *Fusarium keratoplasticum*, were used to prepare ZnO NPs of different shapes [79]. *A. niger* synthesized ZnO nanorods (8–38 nm), and *F. keratoplasticum* synthesized hexagonal NPs (10–42 nm). The shape of the NPs greatly affected their multifunctional properties. Nanorods showed an enhanced antibacterial activity against pathogenic bacteria and a greater UV-protection index, as compared with hexagonal ZnO NPs. Treatment of textile fabrics with a safe dose of green ZnO NPs was potentially active against pathogenic bacteria and improved UV protection, as compared with untreated fabrics.

ZnO NPs obtained with an extract from the zinc-tolerant endophytic fungus *Cochliobolus geniculatus* were polydisperse, non-agglomerated quasi-spherical particles with a narrow range distribution (2–6 nm) [83]. Extracellular proteins involved in the synthesis of ZnO NPs and their capping were also characterized.

A fruit body extract from the medicinal mushroom *Cordyceps militaris* mediated the fabrication of ZnO NPs with an average size of 1.83 nm and with high antimicrobial, antidiabetic, and antioxidant potential [84]. ZnO NPs were also synthesized with a *Daedalea* sp. extract [85]. The NPs were irregularly shaped, had an average size of 14.58 nm, and showed a strong antibacterial and antifungal effect. A *Lentinula edodes* extract was used as a reducing agent involved in the myco-synthesis of ZnO NPs, coated with chitosan, which functions as a stabilizer [86]. The NPs were cubic and hexagonal and had dye-degrading and antibacterial properties.

Quasi-spherical ZnO NPs (16–78 nm) synthesized with a *Periconium* sp. mycelial extract showed good antioxidant properties, as well as excellent antimicrobial effect against fungi, Gram-positive and Gram-negative bacteria [87]. The ability of the yeast *Pichia kudriavzevii* to synthesize ZnO NPs was explored with a fungal extract [88]. The resulting NPs were found to have a hexagonal wurtzite structure with an average crystallite size of ~10–61 nm, and displayed antioxidant and antibacterial properties. The time of the reaction was found to play a critical part in the size, shape, and distribution of NPs. The ability to produce ZnO NPs was also reported for *Pichia fermentans* culture liquid [93].

ZnO NPs synthesized with a *P. djamor* fruit body extract were rod- and cluster-shaped with an average size of 70–80 nm and showed a wide range of biological activities, including larvicidal, antibacterial, antioxidant, and anticancer properties [64]. Semispherical ZnO NPs fabricated with *P. florida* bio-mass varied in size, depending on the precursors [36]. With ZnCl_2_, their size was 21.27–118.36 nm, and with ZnSO_4_, it was 9.36–58.13 nm. In another work, the culture liquid of *Pleurotus floridanus* was used for the synthesis of ZnO nanospheres, and the process parameters were optimized [89]. A *Pleurotus ostreatus* fruit body extract was used to synthesize small (average size of 7.50 nm), spherical, highly stable, agglomerated ZnO NPs with antibacterial and anticancer properties [90].

ZnO NPs with an unusual morphology (fan- and bouquetlike structures 27–40 nm in width and 134–200 nm in length) were fabricated with a *T. harzianum* mycelial extract [33]. In another study, ZnO nanospheres (average size of 30.34 nm) with antioxidant and antibacterial properties were produced with an extract from *T. harzianum* mycelium [91]. *T. viride* culture liquid was also used to synthesize ZnO NPs [92]. These NPs were hexagonal, had an average particle size of about 63.3 nm, and possessed dose-dependent antioxidant activity and appreciable antimicrobial effect against multidrug-resistant microorganisms.

### 2.5. Myco-Synthesis of Nanoparticles of Other Elements

Fungal cultures can form oxides of many elements other than copper, iron, titanium, and zinc. These include magnesium, manganese, cobalt, nickel, zirconium, selenium, tellurium, silicon, cerium, silver, aluminium, bismuth, antimony, gadolinium, and ruthenium (Table 5). Many of these elements remain barely explored in terms of NP myco-synthesis, and their formation by fungal cultures has so far been described only in a few reports.

Several research groups were able to prepare mycogenic NPs of cerium oxide. CeO_2_ nanospheres (5–20 nm) have been successfully obtained with an *A. niger* culture liquid filtrate [97]. The NPs showed a high antibacterial activity against pathogenic bacteria and larvicidal and pupicidal activity against mosquito vectors. Spherical 20–30-nm CeO_2_ NPs obtained with *F. solani* culture liquid showed a good antibacterial and antibiofilm activity against *Psedomonas aeriginosa*, *Klebsiella pneumoniae*, *Escherichia coli*, and *Staphylococcus aureus* [99].

Electrochemical analysis shows that mycogenic NPs can be used in applications such as sensors, batteries, and supercapacitors. The bio-mass of the fungus *Humicola* sp. was able to extracellularly form highly stable, water-dispersible, and highly fluorescent CeO_2_ NPs [100]. These NPs were spherical, were 12–20 nm in diameter, and were naturally capped by proteins secreted by the fungus. Komal et al. synthesized morphologically different CeO_2_ NPs with mycelial extracts from two fungi, *A. terreus* and *Talaromyces prupureogenus* [98]. Pure CeO_2_ NPs synthesized with *A. terreus* were spherical, with an average size of 28.5 nm. CeO_2_ NPs formed by *T. pupureogenus* had a unique nano-sponge morphology and an average size of 21.4 nm. The nano-sponges were more active against *Candida albicans* than were spherical CeO_2_ NPs.

Cobalt oxide NPs have so far been synthesized with several *Aspergillus* species. A cell-free mycelial extract of *Aspergillus brasiliensis* was used to make quasi-spherical monodispersed Co_3_O_4_ NPs of 20–27-nm [101]. The NPs have excellent magnetic properties and show good antimicrobial activity against pathogenic microorganisms. Four fungi were tested by Vijayanandan and colleagues to find a fungus suitable for the synthesis of cobalt oxide NPs [102]. Fluorescent Co_3_O_4_ nanospheres capped with sulfur-bearing proteins were successfully synthesized with *Aspergillus nidulans* bio-mass. Optimization of the fermentation conditions for the enhanced production of Co_3_O_4_ and other NPs with *A. terreus* culture liquid was studied by El-Sayed et al. [29].

Al_2_O_3_ nano-speres with an average size of 30 nm were synthesized with a *Colletotrichum* sp. mycelial extract [94]. Al_2_O_3_ NPs showed a strong antimicrobial activity against food-borne pathogens. Nano-functionalized oil was formulated by combining essential oils from the leaves of *Eucalyptus globulus* and *Citrus medica* with NPs. The combination showed higher antimicrobial activity than did NPs and the essential oils tested separately. Moreover, the activity of commercially available antibiotics increased in combination with mycogenic Al_2_O_3_ NPs.

Irregular silver oxide (AgO) nanospheres of 60–100 nm were synthesized with *A. terreus* culture liquid [95]. The nanospheres had antimicrobial, antioxidant, and antiangiogenic properties. Quasi-spherical bismuth oxide (Bi_2_O_3_) NPs of 5–8 nm were fabricated with *F. oxysporum* bio-mass [96]. Protein-capped, highly stable, and well-dispersed quasi-spherical gadolinium oxide (Gd_2_O_3_) NPs of 3–8 nm were synthesized extracellularly with *Humicola* sp. mycelial bio-mass [103]. These NPs were bio-conjugated with the chemically modified anticancer drug taxol, which may make it more efficient in killing tumor/cancer cells.

Recently, an effective method was described for the fungi-mediated production of fluorescent ruthenium oxide (RuO_2_) QDs by *F. oxysporum* bio-mass [112]. The biosynthesis was conducted under ambient pressure at room temperature, which offers advantages over the previously used chemical and physical methods for RuO_2_ synthesis, requiring highly elevated temperature and pressure. The QDs were monodisperse, non-flocculating, protein capped, and highly stable even months after synthesis. Biosynthesis of antimony trioxide (Sb_2_O_3_) NPs mediated by *S. cerevisiae* was reported by Jha et al. [113]. 

MgO NPs synthesized with *A. niger* culture liquid were nanospheres of 40–95 nm [105]. Their antibacterial activity was stronger against Gram-positive bacteria then it was against Gram-negative ones. Small MgO nanospheres with an average size of 5.8 nm were prepared with an *A. tubingensis* mycelial extract [106]. Larger NPs, of 45.12–95.37 nm, were synthesized with *T. viride* culture liquid and were found to be an effective antibacterial agent [107]. A white button mushroom extract was used to make MgO NPs with an average size of 29.6–38.6 nm [104]. The researchers showed that 16.5- and 15-nm MgO NPs may promote root development in peanut plants.

Quasi-spherical Mn_5_O_8_ NPs of 8–13 nm were obtained with *F. oxysporum* bio-mass [109]. These particles were highly stable, water dispersible, and naturally capped by the fungus-secreted proteins. The Mn-oxidizing fungus *Cladosporium halotolerans* with a strong ability to remove and oxidize Mn (II) was studied by Wang et al. [108]. The *C. halotolerans* culture formed particles with a needlelike or wrinkle-like morphology in the cross section, 2–6 nm in diameter, and 0.1–1 μm in length. The obtained nanowires showed excellent Cd (II) adsorption and had potential anti-pollutant application prospects.

NiO NP synthesis with *A. terreus* culture liquid was optimized for higher NP yield by El-Sayed et al. [29]. The dead bio-mass of the fungus *Hypocrea lixii* was also successfully used to convert nickel ions into NiO NPs in aqueous solution [111]. *H. lixii*–mediated NPs were predominantly spherical and were formed extra- and intracellularly. The NPs formed inside the fungal cells were smaller than those produced extracellularly. The average particle sizes were 3.8 nm for extracellular NPs and 1.25 nm for intracellular NPs. The dead bio-mass of *Aspergillus aculeatus* synthesized spherical NiO NPs in film form [110]. The NPs had a size of about 5.89 nm and were involved in a protein matrix, which probably permitted their organization in film form.

Bansal et al. obtained small quasi-spherical silica NPs with *F. oxysporum* bio-mass. NPs of 5–15 nm were synthesized with K_2_TiF_6_ as a precursor [61]. Moreover, *F. oxysporum* was able to bio-transform naturally occurring amorphous plant bio-silica into crystalline silica and leach out silica extracellularly as 2–6 nm NPs [115]. Spherical silica NPs (40–70 nm) were fabricated extracellularly by an *S. cervisiae* culture [116].

Liang et al. studied the formation of Se- and Te-containing NPs by several fungal species during growth on Se- and Te-containing media [114]. Besides the formation of elementary selenium, selenium oxide was also found after *T. harzianum* had been grown with selenite. Tellurium oxide was detected in the particles generated by *T. harzianum* and *Mortierella humilis* grown with tellurite.

Zirconium oxide (ZrO_2_) nanospheres (40–50 nm in diameter) were fabricated with *F. solani* culture liquid as a reducing and stabilization agent [118]. *F. oxysporum* bio-mass formed quasi-spherical ZrO_2_ NPs (3–11 nm) [117]. Ghomi et al. studied culture liquids of three *Penicillium* species (*Penicillium aculeatum*, *Penicillium notatum*, *Penicillium purpurogenome*) and reported their ability to form ZrO_2_ NPs [119]. All the species studied formed spherical NPs with a size below 100 nm. *Penicillium*-mediated ZrO_2_ NPs showed considerable antibacterial potential against Gram-negative bacteria.

## 3. Myco-Synthesis of Chalcogenide Nanoparticles

Chalcogens are chemical elements from group 16 of the periodic table: oxygen, sulfur, selenium, tellurium, polonium, and livemorioum. Oxygen is often treated separately from the other group 16 elements, or even excluded from the scope of the term “chalcogen”, owing to its very different chemical behavior from the other chalcogens [120]. Chemical compounds consisting of at least one chalcogen anion and at least one more electropositive element are called chalcogenides. The three most important groups of chalcogenides are sulfides, selenides, and tellurides. In recent years, fungi-assisted synthesis of chalcogenide NPs and nanomaterials has become of interest.

### 3.1. Myco-Synthesis of Sulfide Nanoparticles

Fungi-mediated synthesized sulfide NPs are the most commonly studied of all chalcogenides. One of the most important types of semiconductor nanomaterials with a wide band gap and with stability than that of the other chalcogenide NPs, sulfide NPs are more suitable for industrial applications, including high temperature operations, high voltage optoelectronic devices, and as high efficiency electric energy transformers and generators [13].

According to the data so far, fungal cultures can synthesize cadmium, zinc, and lead sulfide NPs. Several researchers have also found that some fungi can produce gold, silver, and copper sulfide NPs (Table 6).

Among mycogenic sulfide NPs, the best studied are cadmium sulfide (CdS) NPs. These were first obtained with the use of fungal cultures in the 1980s, when Dameron and colleagues synthesized quantum CdS nano-crystallites with the yeasts *Candida glabrata* and *Schizosaccharomyces pombe* [154]. Short chelating peptides of general structure (γ-Glu-Cys)_n_-Gly control the nucleation and growth of CdS crystallites to peptide-capped intracellular particles of diameter 20 Å (2 nm). Both yeasts also produce larger, extracellular CdS NPs with an uncharacterized coating, 29 Å (2.9 nm) in diameter. Later, the *S. pombe*–mediated synthesis of CdS NPs was studied by Williams et al. [133]. It was found that intracellular CdS quantum semiconductor crystallites approximately 1.8 nm in diameter can be selectively released from *S. pombe* cells by freezing–thawing. *S. pombe* and *C. glabrata* also formed CdS NPs in their cytoplasm when yeasts were grown in a fed-batch process at high Cd concentrations [124].

*A. niger* bio-mass challenged with precursors produced highly stable poly-dispersed CdS nanospheres with a size ranging from 2.7 to 7.5 nm; these particles were found to have antimicrobial and anticancer abilities [123]. The bio-mass of *F. oxysporum* produced extracellular biocompatible CdS QDs from sulfur waste and Cd(NO_3_)_2_ [126]. These QDs were circular with a diameter of about 6 nm and had a wurtzite crystalline structure. *F. oxysporum* incubated with CdSO_4_ formed CdS NPs in the size range 5–20 nm [125]. *Fusarium* sp. bio-mass, after being exposed to a CdSO_4_ solution, formed spherical CdS NPs with an average particle size of 80 to 120 nm and agglomerates with a size of 350 to 390 nm [127]. CdS QDs synthesized by the fungus *Phanerochaete chrysosporium* had an average size of 2.56 nm [128]. The synthesis of cysteine and proteins was found to play an important part in the formation and stabilization of CdS QDs. CdS QDs with an average size of 8.8 nm formed by *Rhizopus stolonifer* mycelial bio-mass [130]. Luminescent CdS QDs synthesized with *P. ostreatus* mycelium were spherical, were predominantly 4 to 5 nm in size [129], and had anticancer properties [155]. 

*S. cerevisiae*–mediated synthesis of CdS NPs was reported by Prasad and Jha [131]. The NPs were almost spherical, with an average size of 3.57 nm. Another study described the production of CdS nanospheres (average size of 2 nm) with *S. cerevisiae* bio-mass [132]. Cds NPs were formed intracellularly by an *S. pombe* strain [134]. The NPs had a wurtzite (Cd_16_S_20_)-type structure and were mostly in the size range 1–1.5 nm. By using *S. pombe*–mediated CdS NPs, a polymer/nanoparticle diode with a low operating voltage and a high forward current was fabricated. Wurtzite CdS NPs of 3–5 nm were synthesized with a *Termitomyces heimii* mushroom extract [135]. The volume of extract used for the synthesis affected the particle size. A *Trametes versicolor* culture synthesized spherical extracellular CdS QDs with an average size of 6 nm [136]. *T. harzianum* bio-mass formed spherical CdS NPs (3–8 nm) with photocatalytic activity, which was determined upon degradation of methylene blue dye [137]. CdS nanospheres with a size range of about 6–15 nm were formed with the yeast *Trichosporon jirovecii* [138].

Silver sulfide (α-Ag_2_S) NPs were produced with *Humicola* sp. mycelium [121]. These NPs were nanospheres of 15–40 nm and had strong antimicrobial, anticancer, and anti-leishmania activities.

Gold sulfide (Au_2_S) nanospheres with a size of 20–30 nm were synthesized with *Humicola* sp mycelium [122]. The prepared particles were shown to be nontoxic to humans and, therefore, can be proposed for use in leishmania treatment, nano-diagnostics, and drug carrier applications.

Copper sulfide (CuS) NPs were synthesized with *F. oxysporum* mycelium and with CuSO_4_ as a precursor [139]. The formed spherical particles with a size of 2–5 nm were enclosed in spherical peptide shells about 20 nm in diameter. Later, the same authors obtained CuS NPs by incubating *F. oxysporum* mycelium with copper mine wastewater [140]. The results showed that the produced NPs had a covelite composition and that their size was about 10–40 nm.

Lead sulfide (PbS) NPs with quantum semiconductor properties were first synthesized with *Torulopsis* sp. [144]. Seshadri et al. reported the intracellular synthesis of stable PbS NPs by the marine yeast *Rhodosporidium diobovatum* [142]. The NPs, of 2–5 nm, were spherical and well dispersed. *S. cerevisiae* produced PbS nanospheres 0.667–6.95 nm in size [143]. Synthesis of PbS NPs with *A. flavus* yielded 35–100-nm particles that had the potential for the detection of arsenic in aqueous solution [141].

ZnS NPs was synthesized with an extract of the edible mushroom *A. bisporus* [145]. The agglomeration and size of the NPs decreased along with increasing the *A. bisporus* extract volume used for the myco-synthesis. With a high extract volume, the NPs were almost spherical and the average particle size varied from 2.1 to 3.5 nm. The same authors also synthesized ZnS NPs with a *P. ostreatus* fruit body extract [152]. In that case, the agglomeration of the particles and their size also decreased as the amount of the mushroom extract was increased. With the use of a *P. ostreatus* extract, a large quantity of spherical NPs of 4.04 to 2.30 nm was formed. The authors attribute the smaller size of the ZnS NPs obtained with *A. bisporus* to the higher protein content of this mushroom’s fruit bodies [145]. 

Spherical ZnS QDs (average size of 18 nm) were obtained with *A. flavus* bio-mass [146,147]. Later, the same researchers utilized *A. flavus* bio-mass to synthesize gadolinium-doped ZnS (ZnS:Gd) [148]. The fluorescence intensity of the biogenic ZnS:Gd NPs increased in comparison to ZnS NPs, which makes them a reliable fluorescent sensing tool. Jacob and colleagues studied ZnS QD production with bio-mass of two micro-mycetes, *Aspergillus* sp. and *Penicillium* sp. In both cases, the mycogenic NPs were spherical and their average size was 11.08 nm for *Aspergillus* [149] and 6.3 nm for *Penicillium* [151]. *Aspergillus*-mediated ZnS QDs showed excellent antibacterial and dye-degrading activities. The QDs synthesized by *Penicillium* sp. were effective in the photodegradation of methylene blue dye. *F. oxysporum* mycelial bio-mass was used to synthesize spherical ZnS NPs with an mean size of 42 nm [150]. *S. cerevisiae* bio-mass formed 30–40-nm ZnS nanospheres [153].

### 3.2. Myco-Synthesis of Selenide Nanoparticles

Selenides are another important class of chalcogenide semiconductors. Their potential applications include photocatalysis, bioimaging and biolabeling, and nanomedicine [156]. Mycogenic selenide NPs obtained by bio-nanotechnologists so far include cadmium, lead, silver, gold, indium, and neodymium selenides. To date, they have been obtained mostly with fungal bio-mass or living cultures (Table 7).

The most widely studied mycogenic selenide NPs are cadmium selenide (CdSe) NPs, and their synthesis with yeasts is the best studied so far [159,162,163,164,165,170]. Various conditions for CdSe QD synthesis with *S. cerevisiae* were investigated and optimized to obtain particles with a controllable size and with tunable fluorescence emission [163]. Brooks and Lefebvre examined the ability of *S. cerevisiae* sequentially treated with sodium selenite and cadmium chloride to synthesize CdSe QDs in the cytoplasm [164]. They optimized biosynthesis conditions for the highest yield of QDs, and through the optimized method they obtained fluorescent QDs with an average particle diameter of 2.8 nm. Shao et al. studied Se precursors and Se metabolic flux in the synthesis of CdSe QDs in *S. cerevisiae* and improved their ability to synthesize CdSe QDs through gene modification [165]. They identified selenocysteine as the primary Se precursor in the intracellular biosynthesis of CdSe QDs. Further studies showed that the seleno-methionine-to-selenocysteine pathway regulates CdSe QD biosynthesis. Seleno-methionine synthesis was enhanced by overexpression of the *MET6* gene, and the yield of CdSe QDs in the engineered cells was increased.

CdSe QDs (average size of 4.38 nm) were also fabricated in vivo with *Candida utilis* [159]. Higher Cd and lower Se contents favored the formation of QDs with a higher fluorescence intensity and better stability, implying a possibility of tuning the fluorescence properties of these QDs. *Candida*-mediated CdSe QDs were directly used in live-cell imaging without further surface modification. Cao et al. synthesized CdSe QDs with bio-mass of the yeast *Rhodotorula mucilaginosa* [162]. The synthesis was regulated by changing the concentration of precursors and the pH of the medium, and it was found that the concentration of cadmium ions, rather than that of selenium ions, determined the synthesis of CdSe QDs. The synthesized CdSe QDs had a narrow size distribution (3.2 ± 0.4 nm) and great photocatalytic activity toward malachite green dye under ultraviolet and visible light.

Besides various yeasts, other micro-mycetes are also able to form CdSe QDs. *F. oxysporum* bio-mass was used for the synthesis of highly stable semiconductor CdSe QDs (average size of about 11 nm) with a broad fluorescent spectrum [160]. *Helminthosporum solani* mycelial bio-mass induced the synthesis of small monodisperse luminescent CdSe QDs with a mean size of 5.5 nm [161]. Most of these NPs were spherical, and a few cubelike particles were present.

Ag_2_Se QDs with a uniform size of 3.9 nm were obtained with living *S. cerevisiae* yeast [157]. The authors showed that selenocysteine was the primary Se precursor, and the ability to synthesize Ag_2_Se QDs was improved by the construction of engineering strains. Ag_2_Se synthesized with *S. cerevisiae* were weakly toxic and could be used for in vivo imaging.

AuSe nanospheres with an average particle size of 52 nm were synthesized by incubating *F. oxysporum* mycelium with SeCl_4_ and HAuCl_4_ [158]. First, SeCl_4_ was reduced into Se NPs, and then these NPs were reacted with HAuCl_4_ and formed AuSe NPs. In the absence of an Se intermediate, HAuCl_4_ was reduced to Au NPs by fungal biomolecules. AuSe NPs effectively inhibited conidiophores, conidiation, and sporulation in the fungus *A. niger*.

Sinharoy and Lens studied the simultaneous removal of indium, selenium, and tellurium by *A. niger* from media by using different precursor concentrations [166]. Characterization of the fungal bio-mass showed accumulation mostly of elementary Se NPs within mycelial pellets, and, in addition, the formation of indium selenide (InSe) during their simultaneous removal. The NPs were <10 nm in size. Biomimetic fabrication of neodymium selenide (Nd_2_Se_3_) NPs was conducted with a nitrate-dependent reductase from the fungus *F. oxysporum* as a reducing agent and with a synthetic peptide as a capping molecule [167]. Nd_2_Se_3_ NPs were spherical, with an average size of 18 nm.

Fluorescent, semiconductor lead selenide (PbSe) quantum rods were synthesized with the Pb- and Se-tolerant marine fungus *A. terreus* [168]. Semiconductor PbSe NPs were also obtained with *Trichoderma* sp. bio-mass [169]. The optimal ratio between precursors was 1:1 mM SeO_2_:Pb(NO_3_)_2_. The NPs were 10–30-nm cubic face-centered protein-capped particles, which showed a strong antioxidant activity and photocatalytic activity in degrading rhodamine B dye.

### 3.3. Myco-Synthesis of Telluride Nanoparticles

Yet another group of chalcogenides consists of the tellurides, which also show outstanding properties and potential applications in optical, electronic, thermoelectrical, energy storing, catalytic, magnetic, and biological fields [171,172]. Their antimicrobial and anticancer properties allow them to be used in nanomedicine [173]. The fungal synthesis of NPs of these compounds has been poorly studied and has been described by only a few researchers (Table 8).

The ability to fabricate CdTe NPs has been found in several species of micro-mycetes. Highly fluorescent biocompatible CdTe QDs capped with proteins were synthesized with *S. cerevisiae* cells [175]. The synthesized QDs, obtained after 8 days of incubation, were well-dispersed particles with a uniform diameter of about 3.6 nm. When the incubation time was shortened to 2 days, the resulting CdTe QDs were smaller (about 2.2 nm). The CdTe QDs obtained with *F. oxysporum* mycelial bio-mass were also highly fluorescent and were stable and biocompatible [174]. These NPs were spherical, were 15–20 nm in diameter, and showed antibacterial activity against Gram-positive and Gram-negative bacteria. Biocompatible CdTe QDs with an average size of 7.6 nm were also obtained with *R. stolonifer* [133]. The study of the simultaneous removal of indium, selenium, and tellurium by *A. niger* from media showed accumulation of elementary Te NPs and indium telluride NPs within mycelial pellets [166].

## 4. Prospects of Mycogenic Oxide and Chalcogenide Nanoparticles Practical Application

**Figure 1:**
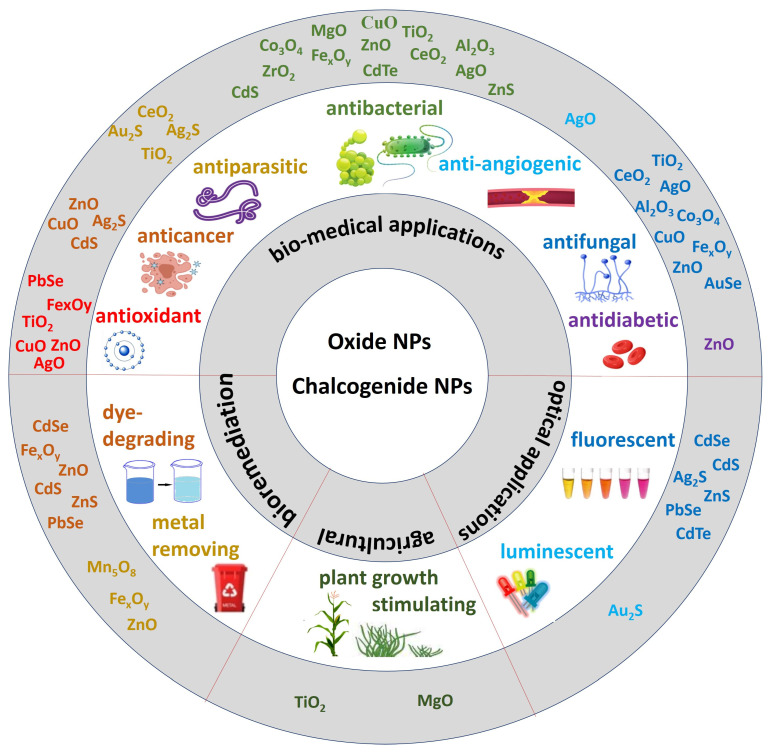
Fungi-mediated oxide and chalcogenide NPs applications.

Many researchers have identified a variety of activities in mycogenic oxide and chalcogenide NPs, determining their great potential for further practical employment in biomedicine, optics, agriculture, pollution control, and other fields of application (Figure 1). 

One of the most important fields for use of these NPs is biomedicine. As described above, various biomedical activities were found in many oxide and chalcogenide NPs produced with fungi. Antibacterial, antifungal, antioxidant, anticancer, antidiabetic, and antileishmanial properties offer great prospects for their use in the treatment and prevention of various diseases. The larvicidal and pupicidal activities against human disease vectors, found in mycogenic TiO_2_ [57], ZnO [64] and CeO_2_ [97] NPs allow them to be used as agents of infection control. The prospects for fungi-derived NP use in agriculture are opened by CuO [33], ZnO [33] and Fe_2_O_3_ [37] NPs’ activity against bacterial and fungal crop pathogens and by TiO_2_ NPs effect against insect pests [70]. Plant growth-promoting properties of TiO_2_ [56] and MgO [104] myco-synthesized NPs enable their use as nano-fertilizers.

Another important characteristic of NPs is their photocatalytic properties, which determine their ability to degrade toxic dyes [176]. Dye-degrading ability was found in ZnO [74,86], ZnS [149,151], CdS [137], CdSe [162] and PbSe [169] mycogenic NPs. In addition, some fungi-derived NPs, such as Fe_2_O_3_ [42] and Fe_3_O_4_ [43], were found to be effective for metal ions removal. These properties allow the use of mycogenic oxide and chalcogenide NPs in the bioremediation of polluted areas and in the treatment of industrial and municipal wastewaters. Thanks to their unique physical and photochemical properties, chalcogenide NPs are actively used in various optical and data storage devices, sensors, batteries and solar cells [120]. Fungi-assisted biosynthesis of these NPs opens up opportunities for environmentally safe NP production for these applications.

## 5. Conclusions

**Figure 2:**
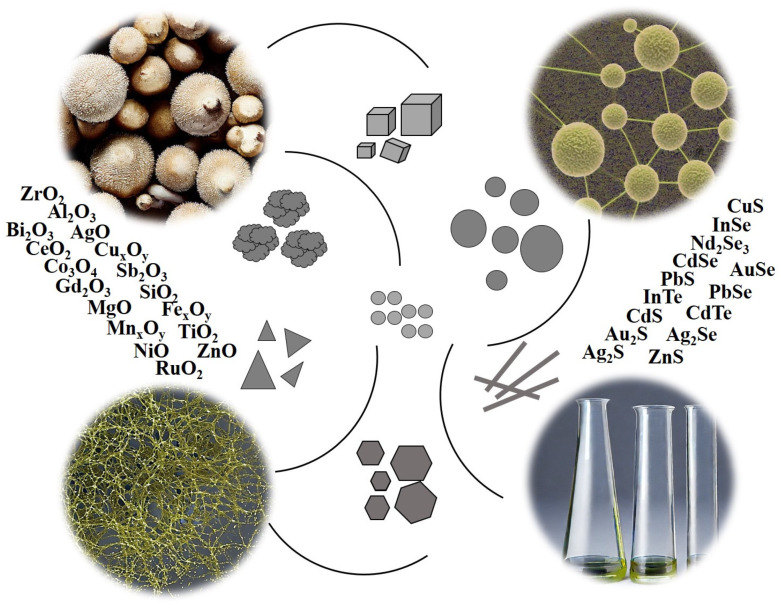
Oxide and chalcogenide NPs biosynthesized by fungi.

As compared with the NPs of elementary metals and metalloids, the myco-synthesis of oxide and chalcogenide NPs is less widely studied, but in recent years, it has attracted increasing research attention. To date, the ability to form oxide and/or chalcogenide NPs has been found in about 70 species of fungi of different taxa, mainly in asco- and basidiomycetes. Fungal cultures can synthesize oxide, sulfide, selenide, and telluride NPs of many elements, which have diverse shapes and sizes (Figure 2).

The characteristics of NPs of the same chemical compound may vary greatly depending on the conditions of their production and are determined not only by the physical and chemical parameters of the reaction (precursors and their concentration, growth medium composition, pH, temperature, stirring rate, lighting, reaction time, and so on) but also by the characteristics of the fungal bio-object used (culture species, strain and age) and by the method of use of the fungus (as a living culture, filtered mycelial bio-mass, spores, extracts from the vegetative mycelium or fruit bodies, cell-free culture liquids, purified metabolites). The study of the influence of all these conditions on NP shape and size, their surface topography, dispersity, stability, aggregation resistance, formation rate, bioavailability, photoluminescent and magnetic properties, and biological activity is very important for selection of the most effective NP producers and optimization of the bio-nano-synthesis methods.

Micro-nano-synthesis of some oxides and chalcogenides, such as TiO2, ZnO, and CdS, has been well studied, but most of the NPs of these compounds remain virtually unexplored in terms of their fungi-mediated production. Fungal cultures are capable of forming NPs of highly diverse chemical compositions, including compounds that are not widely distributed and difficult to obtain, which have great potential for practical applications in various fields of science and technology. Therefore, important challenges facing nano-biotechnologists include:

- screening fungal cultures to identify NP producers of new, previously unexplored compounds;
- further enhancing the knowledge of already known mycogenic oxide and chalcogenide NPs;
- optimization of production methods and scaling up of processes for the biosynthesis of NPs with the required properties on an industrial scale;
- studying the possibilities of practical application of NPs and their introduction into practice.

The enormous potential of fungal cultures as NP producers of various oxides and chalcogenides, together with the extremely poor state of knowledge of the myco-nano-synthesis of the majority of these NPs, makes their further detailed and in-depth study, as objects for the biosynthesis of the oxide and chalcogenide NPs and nanomaterials based on them, a highly important task.

## Figures and Tables

**Table 1:** Myco-synthesis of copper oxide nanoparticles.

NP	Species	Source	Precursors	Shape and Size	Reference
CuO	*Aspergillus flavus*	Culture liquid	CuSO_4_	Spherical (average size of 32.4 nm)	[27]
CuO	*Aspergillus terreus*	Culture liquid	CuSO_4_	–	[28,29]
CuO	*Aspergillus terreus*	Culture liquid	CuSO_4_	Below 100 nm	[30]
CuO	*Penicillium chrysogenum*	Culture liquid	CuSO_4_	Spherical (average size of 9.7 nm)	[31]
CuO	*Trichoderma asperellum*	Mycelial extract	Cu(NO_3_)_2_	Spherical (10–190 nm)	[32]
CuO	*Trichoderma harzianum*	Mycelial extract	CuSO_4_	Nano-fibers (38–77 nm in width, 135–320 nm in length)	[33]
Cu_2_O, CuO	*Stereum hirsutum*	Mycelial extract	CuCl_2_	Spherical (5–20 nm)	[34]
Cu_x_O_y_	*Penicillium aurantiogriseum*	Culture liquid	CuSO_4_	Spherical (89–250 nm)	[35]
Cu_x_O_y_	*Penicillium citrinum*	Culture liquid	CuSO_4_	Spherical (85–295 nm)	[35]
Cu_x_O_y_	*Penicillium waksmanii*	Culture liquid	CuSO_4_	Spherical (79–179 nm)	[35]
Cu_x_O_y_	*Pleurotus florida*	Bio-mass	CuCl_2_	Spherical, partially spherical, oval (22.55–60.09 nm)	[36]
			CuSO_4_	Hexagonal, partially spherical (12.82–48.86 nm)	

**Table 2:** Myco-synthesis of iron oxide nanoparticles.

NP	Species	Source	Precursors	Shape and Size	Reference
α-Fe_2_O_3_	*Trichoderma harzianum*	Mycelial extract	FeCl_3_	Spherical (average size of 207 nm)	[37]
γ-Fe_2_O_3_	*Penicillium expansum*	Mycelial extract	FeCl_3_	Spherical (15.0–66.0 nm)	[38]
γ-Fe_2_O_3_/α-Fe_2_O_3_	*Alternaria alternata*	Mycelial extract	Fe(NO_3_)_3_	Cubic (average size of 9 nm)	[39]
Fe_2_O_3_	*Aspergillus niger*	Bio-mass	FeCl_3_	–	[40]
Fe_2_O_3_	*Fusarium incarnatum*	Culture liquid	FeCl_2_ + FeCl_3_	Spherical (average size of 30.56 nm)	[41]
Fe_2_O_3_	*Phialemoniopsis ocularis*	Culture liquid	FeCl_2_ + FeCl_3_	Spherical (average size of 13.13 nm)	[41]
Fe_2_O_3_	*Penicillium pimiteouiense*	Culture liquid	FeCl_2_ + FeCl_3_	Spherical (2–16 nm)	[42]
Fe_2_O_3_	*Trichoderma asperellum*	Culture liquid	FeCl_2_ + FeCl_3_	Spherical (average size of 25 nm)	[41]
Fe_3_O_4_	*Aspergillus niger*	Mycelial extract	FeCl_3_	Nanoflakes (20–40 nm)	[43]
Fe_3_O_4_	*Aspergillus terreus*	Culture liquid	Fe(NO_3_)_3_	Spherical	[28,29]
Fe_3_O_4_	*Aspergillus tamarii*	Culture liquid	FeSO_4_ + FeCl_3_	Spherical (5–22 nm)	[44]
Fe_3_O_4_	*Fusarium oxysporum*	Bio-mass	K_3_[Fe(CN)_6_] + K_4_[Fe(CN)_6_]	Quasi-spherical (20–50 nm)	[45]
Fe_3_O_4_	*Fusarium solani*	Bio-mass	Fe_2_O_3_	Cubic, spherical, irregular (55.3–84.2 nm)	[46]
Fe_3_O_4_	*Verticillium* sp.	Bio-mass	K_3_[Fe(CN)_6_] + K_4_[Fe(CN)_6_]	Cubo-octahedral (100–400 nm)	[45]
Fe_x_O_y_	*Amanita muscaria*	Fruit body extract	FeCl_2_ + FeCl_3_	2.2–2.5 nm	[47]
Fe_x_O_y_	*Aspergillus japonicus*	Bio-mass	K_3_[Fe(CN)_6_] + K_4_[Fe(CN)_6_]	Cubic (60–70 nm)	[48]
Fe_x_O_y_	*Pleurotus florida*	Bio-mass	FeCl_2_	Cubic (11.90–167.63 nm)	[36]
			FeSO_4_	Spherical (11.16–98.81 nm), highly agglomerated	

**Table 3:** Myco-synthesis of titanium oxide nanoparticles.

NP	Species	Source	Precursors	Shape and Size	Reference
TiO_2_	*Agaricus bisporus*	Spores	Ti(OC_3_H_7_)_4_	–	[54]
TiO_2_	*Aspergillus flavus*	Bio-mass	TiO_2_	Spherical, oval (62–74 nm)	[55]
TiO_2_	*Aspergillus flavus*	Mycelial extract	TiO_2_	12–15 nm	[56]
TiO_2_	*Aspergillus niger*	Mycelial extract	TiO_2_ salt	Spherical (73.58–106.9 nm)	[57]
TiO_2_	*Aspergillus tubingensis*	Mycelial extract	TiO_2_ salt	Cubic, pentangular (1.5–30 nm)	[58]
TiO_2_	*Fomes fomentarius*	Fruit body extract	Ti{OCH(CH_3_)_2_}_4_	Irregular (80–120 nm)	[59]
TiO_2_	*Fomitopsis pinicola*	Fruit body extract	Ti{OCH(CH_3_)_2_}_4_	Irregular (80–120 nm)	[60]
TiO	*Fusarium oxysporum*	Bio-mass	K_2_TiF_6_	Spherical (6–13 nm)	[61]
TiO_2_	*Humicola* sp.	Bio-mass	Bulk TiO_2_	Spherical (5–28 nm)	[62]
TiO_2_	*Hypsizygus ulmarius*	Fruit body extract	TiCl_4_	Spherical (average size of 80 nm)	[63]
TiO_2_	*Pleurotus djamor*	Fruit body extract	TiCl_4_	Spherical (average size of 31 nm)	[64]
TiO_2_	*Pleurotus sajor caju*	Fruit body extract	TiCl_4_	Spherical (average size of 85 nm)	[65]
TiO_2_	*Sachharomyces cerevisae*	Living culture	TiO(OH)_2_	Spherical (average size of 12.57 nm)	[66]
TiO_2_	*Sachharomyces cerevisae*	Living culture	TiCl_4_	Oval (10–12 nm), mesoporous	[67]
TiO_2_	*Sachharomyces cerevisae*	Living culture	TiCl_3_	Spherical (average size of 6.7 nm)	[68]
TiO_2_	*Tricoderma citrinoviride*	Mycelial extract	Ti{OCH(CH_3_)_2_}_4_	Irregular, triangular, pentagonal, spherical, rod-shaped (10–400 nm)	[69]
TiO_2_	*Trichoderma viride*	Culture liquid	TiO(OH)_2_	Spherical (60–86.67 nm)	[70]

**Table 4:** Myco-synthesis of zinc oxide nanoparticles.

NP	Species	Source	Precursors	Shape and Size	Reference
ZnO	*Acremonium potronii*	Mycelial extract	Zn(CH_3_CO_2_)_2_	Spherical (13–15 nm)	[74]
ZnO	*Agarius bisporus*	Fruit body extract	Zn(CH_3_CO_2_)_2_	Spherical (average size of 14.48 nm)	[75]
ZnO	*Aspergillus aeneus*	Mycelial extract	Zn(CH_3_CO_2_)_2_	Spherical (100–140 nm)	[76]
ZnO	*Aspergillus fumigatus*	Culture liquid	ZnSO_4_	Spherical (60–80 nm)	[77]
ZnO	*Aspergillus fumigatus*	Mycelial extract	Zn(NO_3_)_2_	Oblate spherical and hexagonal (1.2–6.8 nm)	[78]
ZnO	*Aspergillus niger*	Mycelial extract	Zn(CH_3_CO_2_)_2_	Nanorods (8–38 nm)	[79]
ZnO	*Aspergillus niger*	Crushed fungal powder	ZnCl_2_	Hexagonal (average size of 66 nm)	[80]
ZnO	*Aspergillus terreus*	Culture liquid	ZnSO_4_	Spherical (28–63 nm)	[81]
ZnO	*Aspergillus terreus*	Mycelial extract	Zn(CH_3_CO_2_)_2_	Spherical (10–45 nm)	[82]
ZnO	*Aspergillus terreus*	Culture liquid	ZnC_4_H_6_O_4_	Almost spherical	[28,29]
ZnO	*Cochliobolus geniculatus*	Mycelial extract	Zn(CH_3_CO_2_)_2_	Quasi-spherical (2–6 nm)	[83]
ZnO	*Cordyceps militaris*	Fruit body extract	Zn(CH_3_CO_2_)_2_	Spherical, irregular (average size of 1.83 nm)	[84]
ZnO	*Daedalea* sp.	Fruit body extract	Zn(CH_3_CO_2_)_2_	Irregular (average size of 14.58 nm)	[85]
ZnO	*Fusarium keratoplasticum*	Mycelial extract	Zn(CH_3_CO_2_)_2_	Hexagonal (10–42 nm)	[79]
ZnO	*Lentinula edodes*	Fruit body extract	Zn(NO_3_)_2_	Cubic, hexagonal (average size of 50 nm)	[86]
ZnO	*Periconium* sp.	Mycelial extract	Zn(NO_3_)_2_	Quasi-spherical (16–78 nm)	[87]
ZnO	*Pichia kudriavzevii*	Fungal extract	Zn(CH_3_CO_2_)_2_	Hexagonal (average size of 32 nm)	[88]
ZnO	*Pleurotus djamor*	Fruit body extract	Zn(NO_3_)_2_	Nanorods, clusters (average size of 70–80 nm)	[64]
ZnO	*Pleurotus florida*	Bio-mass	ZnCl_2_	Semi-spherical (21.27–118.36 nm)	[36]
			ZnSO_4_	Semi-spherical (9.36–58.13 nm)	
ZnO	*Pleurotus floridanus*	Culture liquid	Zn(NO_3_)_2_	Spherical (average size of 34.98 nm)	[89]
ZnO	*Pleurotus ostreatus*	Fruit body extract	Zn(NO_3_)_2_	Spherical (average size of 7.50 nm)	[90]
ZnO	*Trichoderma harzianum*	Mycelial extract	ZnSO_4_	Fan- and bouquet-like structures (27–40 nm in width, 134–200 nm in length)	[33]
ZnO	*Trichoderma harzianum*	Mycelial extract	Zn(CH_3_CO_2_)_2_	Spherical (average size of 30.34 nm)	[91]
ZnO	*Trichoderma viride*	Culture liquid	Zn(CH_3_CO_2_)_2_	Hexagonal (average size of 63.3 nm)	[92]

**Table 5:** Myco-synthesis of nanoparticles of other elements.

NP	Species	Source	Precursors	Shape and Size	Reference
Al_2_O_3_	*Colletotrichum* sp.	Mycelial extract	AlCl_3_	Spherical (average size of 30 nm)	[94]
AgO	*Aspergillus terreus*	Culture liquid	AgNO_3_	Irregular spherical (60–100 nm)	[95]
Bi_2_O_3_	*Fusarium oxysporum*	Bio-mass	Bi(NO_3_)_3_	Quasi-spherical (5–8 nm)	[96]
CeO_2_	*Aspergillus niger*	Culture liquid	CeCl_3_	Spherical (5–20 nm)	[97]
CeO_2_	*Aspergillus terreus*	Mycelial extract	Ce(NO_3_)_3_	Spherical (average size of 28.5 nm)	[98]
CeO_2_	*Fusarium solani*	Culture liquid	CeCl_3_	Spherical (20–30 nm)	[99]
CeO_2_	*Humicola* sp.	Bio-mass	Ce(NO_3_)_3_	Spherical (12–20 nm)	[100]
CeO_2_	*Talaromyces prupureogenus*	Mycelial extract	Ce(NO_3_)_3_	Nano-sponges (average size of 21.4 nm)	[98]
Co_3_O_4_	*Aspergillus brasiliensis*	Mycelial extract	CoSO_4_	Quasi-spherical (20–27 nm)	[101]
Co_3_O_4_	*Aspergillus nidulans*	Bio-mass	Co(C_5_H_7_O_2_)_2_	Spherical (average size of 20.29 nm)	[102]
Co_3_O_4_	*Aspergillus terreus*	Culture liquid	CoSO_4_	Spherical	[28,29]
Gd_2_O_3_	*Humicola* sp.	Bio-mass	GdCl_3_	Quasi-spherical (3–8 nm)	[103]
MgO	*Agaricus bisporus*	Fruit body extract	Mg(CH_3_COO)_2_	29.6–38.6 nm	[104]
MgO	*Aspergillus niger*	Culture liquid	MgCl_2_	Spherical (40–95 nm)	[105]
MgO	*Aspergillus tubingensis*	Mycelial extract	Mg(NO_3_)_2_	Spherical (average size of 5.8 nm)	[106]
MgO	*Trichoderma viride*	Culture liquid	MgCl_2_	45.12–95.37 nm	[107]
Mn_x_O_y_	*Cladosporium halotolerans*	Living culture	MnCl_2_	Needle-like (2–6 nm in diameter, 0.1–1 μm in length)	[108]
Mn_5_O_8_	*Fusarium oxysporum*	Bio-mass	(CH_3_CO_2_)_2_Mn·	Quasi-spherical (8–13 nm)	[109]
NiO	*Aspergillus aculeatus*	Dead bio-mass	NiCl_2_	Spherical (average size of 5.89 nm)	[110]
NiO	*Aspergillus terreus*	Culture liquid	NiSO_4_	Spherical	[28,29]
NiO	*Hypocrea lixii*	Dead bio-mass	NiCl_2_	Average size of 3.8 nm for extracellular and 1.25 nm for intracellular NPs	[111]
RuO_2_	*Fusarium oxysporum*	Bio-mass	RuCl_3_	Spherical (2–5 nm)	[112]
Sb_2_O_3_	*Saccharomyces cerevisiae*	Fungal culture	SbCl_3_	Spherical (2–10 nm)	[113]
SeO_2_	*Trichoderma harzianum*	Living culture	Na_2_SeO_3_	–	[114]
SiO_2_	*Fusarium oxysporum*	Bio-mass	K_2_SiF_6_	Quasi-spherical (5–15 nm)	[61]
SiO_2_	*Fusarium oxysporum*	Bio-mass	Amorphous silica present in rice husk	Quasi-spherical (2–6 nm)	[115]
SiO_2_	*Saccharomyces cervisiae*	Living culture	Sodium silicate	Spherical (40–70 nm)	[116]
TeO_2_	*Mortierella humilis*	Living culture	Na_2_TeO_3_	–	[114]
TeO_2_	*Trichoderma harzianum*	Living culture	Na_2_TeO_3_	–	[114]
ZrO_2_	*Fusarium oxysporum*	Bio-mass	K_2_ZrF_6_	Quasi-spherical (3–11 nm)	[117]
ZrO_2_	*Fusarium solani*	Culture liquid	zirconyl nitrate	Spherical (40–50 nm)	[118]
ZrO_2_	*Penicillium aculeatum*	Culture liquid	ZrCl_4_	Spherical (average size of 39.32 nm)	[119]
ZrO_2_	*Penicillium notatum*	Culture liquid	ZrCl_4_	Spherical (average size of 62.27 nm)	[119]
ZrO_2_	*Penicillium purpurogenome*	Culture liquid	ZrCl_4_	Spherical (average size of 53.60 nm)	[119]

**Table 6:** Myco-synthesis of sulfide nanoparticles.

NP	Species	Source	Precursors	Shape and Size	Reference
α-Ag_2_S	*Humicola* sp.	Mycelial bio-mass	AgNO_3_, Na_2_SO_3_	Spherical (15–40 nm)	[121]
Au_2_S	*Humicola* sp.	Mycelial bio-mass	HAuCl_4_, Na_2_SO_3_	Spherical (20–30 nm)	[122]
CdS	*Aspergillus niger*	Mycelial bio-mass	CdCl_2_, Na_2_S	Spherical (2.7–7.5 nm)	[123]
CdS	*Candida glabrata*	Living culture	Cd(NO_3_)_2_	–	[124]
CdS	*Fusarium oxysporum*	Living culture	CdSO_4_	5–20 nm	[125]
CdS	*Fusarium oxysporum*	Mycelial bio-mass	Cd(NO_3_)_2_, sulfur waste	Spherical (average size of 6 nm)	[126]
CdS	*Fusarium* sp.	Mycelial bio-mass	CdSO_4_	Spherical (80–120 nm)	[127]
CdS	*Phanerochaete chrysosporium*	Living culture	Cd(NO_3_)_2_	Average size of 2.56 nm	[128]
CdS	*Pleurotus ostreatus*	Mycelial bio-mass	CdSO_4_, Na_2_S	Spherical (4–5 nm)	[129]
CdS	*Rhizopus stolonifer*	Mycelial bio-mass	CdCl_2_, ZnS	Average size of 8.8 nm	[130]
CdS	*Saccharomyces cerevisiae*	Living culture	CdS solution	Spherical (average size of 3.57 nm)	[131]
CdS	*Saccharomyces cerevisiae*	Bio-mass	CdCl_2_, Na_2_S	Spherical (average size of 2 nm)	[132]
CdS	*Schizosaccharo-myces pombe*	Living culture	CdSO_4_	Average size of 1.8 nm	[133]
CdS	*Schizosaccharo-myces pombe*	Living culture	CdSO_4_	1–1.5 nm	[134]
CdS	*Schizosaccharo-myces pombe*	Living culture	Cd(NO_3_)_2_	–	[124]
CdS	*Termitomyces heimii*	Fruit body extract	Cd(NO_3_)_2_, Na_2_S	Spherical (3–5 nm)	[135]
CdS	*Trametes versicolor*	Living culture	Cd(NO_3_)_2_	Spherical (average size of 6 nm)	[136]
CdS	*Trichoderma harzianum*	Mycelial bio-mass	CdCl_2_, Na_2_S	Spherical (3–8 nm)	[137]
CdS	*Trichosporon jirovecii*	Living culture	CdCl_2_	Spherical (6–15 nm)	[138]
CuS	*Fusarium oxysporum*	Mycelial bio-mass	CuSO_4_	Spherical (2–5 nm)	[139]
CuS	*Fusarium oxysporum*	Mycelial bio-mass	Copper mine wastewaters	10–40 nm	[140]
PbS	*Aspergillus flavus*	Living culture	Pb(C_2_H_3_O_2_)_2_, Na_2_S	35–100 nm	[141]
PbS	*Rhodosporidium diobovatum*	Bio-mass	Pb(NO_3_)_2_	Spherical (2–5 nm)	[142]
PbS	*Saccharomyces cerevisiae*	Living culture	Pb(C_2_H_3_O_2_)_2_, Na_2_S	Spherical (0.667–6.95 nm)	[143]
PbS	*Torulopsis* sp.	Living culture	Pb(NO_3_)_2_	2–5 nm	[144]
ZnS	*Agaricus bisporus*	Fruit body extract	ZnCl_2_, Na_2_S	Almost spherical (2.1–3.5 nm)	[145]
ZnS	*Aspergillus flavus*	Mycelial bio-mass	ZnSO_4_	Spherical (average size of 18 nm)	[146,147]
ZnS:Gd	*Aspergillus flavus*	Mycelial bio-mass	ZnSO_4_, Gd(NO_3_)_2_	Spherical (10–18 nm)	[148]
ZnS	*Aspergillus* sp.	Mycelial bio-mass	ZnSO_4_	Spherical (average size of 11.08 nm)	[149]
ZnS	*Fusarium oxysporum*	Mycelial bio-mass	ZnSO_4_	Spherical (average size of 42 nm)	[150]
ZnS	*Penicillium* sp.	Mycelial bio-mass	ZnSO_4_	Spherical (average size of 6.3 nm)	[151]
ZnS	*Pleurotus ostreatu*	Fruit body extract	ZnCl_2_, Na_2_S	Almost spherical (2.1–3.5 nm)	[152]
ZnS	*Saccharomyces cerevisiae*	Bio-mass	ZnSO_4_	Spherical (30–40 nm)	[153]

**Table 7:** Myco-synthesis of selenide nanoparticles.

NP	Species	Source	Precursors	Shape and Size	Reference
Ag_2_Se	*Saccharomyces cerevisiae*	Living culture	AgNO_3_, Na_2_SeO_3_	Average size of 3.9 nm	[157]
AuSe	*Fusarium oxysporum*	Mycelial bio-mass	HAuCl_4_, SeCl_4_	Spherical (average size of 52 nm)	[158]
CdSe	*Candida utilis*	Living culture	CdCl_2_, Na_2_SeO_3_	Average size of 4.38 nm	[159]
CdSe	*Fusarium oxysporum*	Mycelial bio-mass	CdCl_2_, SeCl_4_	Average size of 11 nm	[160]
CdSe	*Helminthosporum solani*	Mycelial bio-mass	CdCl_2_, SeCl_4_	Spherical, cubic (average size of 5.5 nm)	[161]
CdSe	*Rhodotorula mucilaginosa*	Bio-mass	CdCl_2_, Na_2_SeO_3_	Average size of 3.2 nm	[162]
CdSe	*Saccharomyces cerevisiae*	Living culture	CdCl_2_, Na_2_SeO_3_	15–20 nm	[163]
CdSe	*Saccharomyces cerevisiae*	Living culture	CdCl_2_, Na_2_SeO_3_	Average size of 2.8 nm	[164]
CdSe	*Saccharomyces cerevisiae*	Bio-mass	CdCl_2_, Na_2_SeO_3_	–	[165]
InSe	*Aspergillus niger*	Living culture	InCl_3_, Na_2_SeO_3_	<10 nm	[166]
Nd_2_Se_3_	*Fusarium oxysporum*	Fungal nitrate-dependent reductase	NdCl_2_, SeCl_4_	Spherical (average size of 18 nm)	[167]
PbSe	*Aspergillus terreus*	–	–	Nanorods (average size of 59 nm)	[168]
PbSe	*Trichoderma* sp.	Mycelial bio-mass	Pb(NO_3_)_2_, SeO_2_	Cubic (10–30 nm)	[169]

**Table 8:** Myco-synthesis of telluride nanoparticles.

NP	Species	Source	Precursors	Shape and Size	Reference
CdTe	*Fusarium oxysporum*	Mycelial bio-mass	CdCl_2_, TeCl_4_	Spherical (15–20 nm)	[174]
CdTe	*Rhizopus stolonifer*	Mycelial bio-mass	CdCl_2_, TeCl_4_	QDs (average size of 7.6 nm)	[133]
CdTe	*Saccharomyces cerevisiae*	Living culture	CdCl_2_, Na_2_TeO_3_	QDs (2.0–3.6 nm)	[175]
InTe	*Aspergillus niger*	Living culture	InCl_3_, K_2_TeO_3_	<10 nm	[166]

## Data Availability

Not applicable.
